# Supplementary material for: Determinants of low birth weight among newborns delivered in public hospitals of North Shewa Zone, Amhara region, Ethiopia: A case-control study (2023)
Source: PLoS One. 2024 May 13;19(5):e0303364. doi: 10.1371/journal.pone.0303364 (PMC11090310; doi:10.1371/journal.pone.0303364)
Supplement: Attachment — Submitted filename: Response to Reviewers_1 Bnyas.docx [file pone.0303364.s002.docx]

| Comments given | Area of comments | Responses | Remarks |
| --- | --- | --- | --- |
| Academic Editor | Over all   - •Please revise the introduction section as commented by Reviewer 1. Please try to synthesize and summarize in too few paragraphs. It has to be shortened but brief, precise, and comprehensive enough considering the available literatures - The methods section needs much revision as detail description (should be comprehensive but summary) of the methods used is very important to replicate the study elsewhere. Ambiguities and unclear concepts should be clarified. For detail refer to the reviewers' comments. - • As indicated by a reviewer, the statistical analysis has not been rigorously performed. Please clarify this. Please describe the results in detail, particularly the determinants of LBW, especially results from multivariable logistic regression model - As discussion is not only comparing your main findings with that of other previously conducted studies, please try to include the implication of the main findings in addition to explaining the reason of the gaps between discrepancies of the findings (i.e. for lower and higher results). - Please include your tables as part of your main manuscript and remove the individual files. Please note that supplementary tables (should remain/ be uploaded) as separate "supporting information" files - Please include captions for your Supporting Information files at the end of your manuscript, and update any in-text citations to match accordingly. | Accepted and corrected |  |
| Reviewer #1 | Background  -You can merge the 2nd and the 3rd paragraph because they both are talking about the magnitude of LBW.  Generally, you described background information for manuscript in more than 10 paragraphs (some of them are incomplete with two or three sentences). Please try to synthesize and summarize in four paragraphs.  -Please bring sentences talking about the significance of your study together, than talking about significance of the study in many places. | Accepted and corrected |  |
|  | Method section;You said, four hospitals were selected from 11 hospitals. But it looks that the selected hospitals are more than 4 (1 comprehensive, 1 teaching, 2 general, and the rest primary) from the following statement taken from your manuscript: The selected hospitals included one comprehensive specialized governmental hospital, one teaching hospital, two general hospitals, and the rest being primary hospitals.  -Please explain if you have any justification for including only term babies or why preterm excluded?  -One of your inclusion criteria for controls says term singleton live-births with a birth weight >2500g. What if birth weight is greater than 4000g?  -Please do not mention preterm as an exclusion criterion because it is by default excluded in your inclusion criteria  -Please explain what does new-borns with multiple births mean (it is one of your exclusion criteria)  -Please provide any justification for excluding diabetic mothers.  -Please provide more clarification on how you selected controls using systematic random sampling?  -You have stated about informed consent under data collection method. Please take it to the ethics section.  -Please add statistical analysis methods you went through | Accepted and corrected |  |
|  | Result ; Please add reports related to determinants of LBW, especially results from multivariable logistic regression model  Please add conclusion | Accepted and corrected |  |
| Reviewer #2: | Methods ;Way of sample size determination is not clear, make it clear.  *You used P-Value 0.25 for binary logistic regression, justify it how it can be. | Accepted and corrected | It is calculated by using Epi Info software. |
|  | Result ; There are no variables determined under result part. Which are significantly associated with dependent variables after entered into Multivariable logistic regression model? Put such variables in result part and explain them. | Accepted and corrected |  |
|  | Discussion, there is visible comparison between the current and previous study and also explain the reason of the gaps between current and previous study | Accepted and corrected |  |
|  | Conclusion ;There is no conclusion without the variable's determination on last (multivariable logistic regression), so before conclusion you should identify the variables significantly associated by multivariable logistic regression | Accepted and corrected |  |
