# Supplementary material for: Determinants of low birth weight among newborns delivered in public hospitals of North Shewa Zone, Amhara region, Ethiopia: A case-control study (2023)
Source: PLoS One. 2024 May 13;19(5):e0303364. doi: 10.1371/journal.pone.0303364 (PMC11090310; doi:10.1371/journal.pone.0303364)
Supplement: Attachment — Submitted filename: Review Feedback.docx [file pone.0303364.s003.docx]

**Still the majority of comments were not corrected and responded as they were commented.**

*You used P-Value 0.25 for binary logistic regression, justify it how it can be.
* There are no variables determined under result part. Which are significantly associated with dependent variables after entered into Multivariable logistic regression model? Put such variables in result part and explain them.
* There is no conclusion without the variable's determination on last (multivariable logistic regression), so before conclusion you should identify the variables significantly associated by multivariable logistic regression.
