# Supplementary material for: Determinants of low birth weight among newborns delivered in public hospitals of North Shewa Zone, Amhara region, Ethiopia: A case-control study (2023)
Source: PLoS One. 2024 May 13;19(5):e0303364. doi: 10.1371/journal.pone.0303364 (PMC11090310; doi:10.1371/journal.pone.0303364)
Supplement: Attachment — Submitted filename: Response to Reviewers_3.docx [file pone.0303364.s004.docx]

Response to Reviewers

| Comments given by | Area of comments | Comment given | Response given by Authors |
| --- | --- | --- | --- |
| Reviewer | Result | You used P-Value 0.25 for binary logistic regression, justify it how it can be | We did not utilize a p-value threshold of less than 0.25 for making statistical assertions. Instead, we employed this threshold as a screening criterion for selecting variables in multivariable logistic regression, aiming to streamline the candidate variable pool. For determining statistical significance, we adhered to the conventional threshold of less than 0.05. |
|  |  | There are no variables determined under result part. | Accepted and corrected |
|  |  | There is no conclusion | Accepted and corrected |
