# Supplementary material for: Determinants of low birth weight among newborns delivered in public hospitals of North Shewa Zone, Amhara region, Ethiopia: A case-control study (2023)
Source: PLoS One. 2024 May 13;19(5):e0303364. doi: 10.1371/journal.pone.0303364 (PMC11090310; doi:10.1371/journal.pone.0303364)
Supplement: Attachment — Submitted filename: Response to Reviewers_4.docx [file pone.0303364.s005.docx]

Response to Reviewers

| Comments given by | Area of comments | Comment given | Response given by Authors | Remark |
| --- | --- | --- | --- | --- |
| Reviewer | Result | You used P-Value 0.25 for binary logistic regression, justify it how it can be | We did not utilize a p-value threshold of less than 0.25 for making statistical assertions. Instead, we employed this threshold as a screening criterion for selecting variables in multivariable logistic regression, aiming to streamline the candidate variable pool. For determining statistical significance, we adhered to the conventional threshold of less than 0.05. | Accepted and corrected |
|  |  | There are no variables determined under result part. | Furthermore, the study investigated various factors associated with low birth weight at a significance level of p < 0.05. Among newborns, females exhibited a higher risk, with an adjusted odds ratio (AOR) of 3.13 (95% CI: 1.34, 7.32, p = 0.008), while mothers aged 20 or younger had an AOR of 3.42 (95% CI: 1.35, 8.66, p = 0.009). Notably, individuals with no formal education faced a substantially increased risk (AOR: 6.82, 95% CI: 2.94, 15.3, p < 0.001). Additionally, unplanned pregnancies were associated with a higher risk (AOR: 3.08, 95% CI: 1.38, 6.84, p = 0.006), as were instances where mothers did not attend antenatal care (AOR: 2.74, 95% CI: 1.16, 6.49, p = 0.021). Conversely, factors such as residency type and maternal age above 35 did not demonstrate significant associations | Accepted and corrected |
|  |  | There is no conclusion | **Conclusion and Recommendations:**  The study highlights the urgent need to address LBW in North Shewa Zone, Amhara Region, Ethiopia, where determinants like maternal age ≤ 20 years and inadequate dietary diversity pose significant risks. Enhanced education, strengthened antenatal care, nutritional support, and community interventions are recommended. Policy initiatives advocating for improved maternal education and healthcare access are vital. Implementing these strategies will mitigate LBW incidence and improve maternal and child health outcomes in the region**.** | Accepted and corrected **;** |
